# Blei im Trinkwasser – ein altes Problem, eine neue EU-Richtlinie

**DOI:** 10.1007/s00103-021-03292-2

**Published:** 2021-02-26

**Authors:** Manuel Döhla, Andreas Jaensch, Christin Döhla, Alexander Voigt, Martin Exner, Harald Färber

**Affiliations:** 1 Institut für Hygiene und Öffentliche Gesundheit, Universitätsklinikum Bonn, Venusberg-Campus 1, 53127 Bonn, Deutschland; 2 Abteilung für Mikrobiologie und Krankenhaushygiene, Bundeswehrzentralkrankenhaus Koblenz, Koblenz, Deutschland; 3 Postgraduales Studium „Toxikologie und Umweltschutz“, Institut für Rechtsmedizin, Universität Leipzig, Leipzig, Deutschland

**Keywords:** Blei, Exposition, Trinkwasser, EU-Richtlinie, Grenzwert, Lead, Exposure, Drinking water, EU directive, Limit value

## Abstract

**Hintergrund:**

Bleirohre wurden lange Zeit in Trinkwasserinstallationen verbaut, erst seit 1973 wird von ihrer Verwendung in Neubauten dringend abgeraten; dennoch finden sie sich noch in alten Gebäuden. Bleihaltige Legierungen werden daneben bis heute in Bauteilen wie Armaturen verwendet. So resultiert eine vermeidbare Belastung des Trinkwassers. Die gesundheitliche Bedeutung dieser Belastung wird mit einer Senkung des derzeit geltenden Grenzwertes von 10 µg/l auf 5 µg/l durch die 2020 verabschiedete neue EU-Trinkwasserrichtlinie gewürdigt. Diese sieht eine Übergangsfrist von 15 Jahren vor.

**Ziel der Arbeit:**

Die Relevanz eines strengeren Grenzwertes für Blei im Trinkwasser soll anhand der Ergebnisse von Routineanalysen bewertet werden sowie notwendige Public-Health-Maßnahmen zur Expositionsprophylaxe vulnerabler Gruppen sollen formuliert werden.

**Material und Methoden:**

Es wurde eine retrospektive Analyse von Routineproben aus der Stadt Bonn durchgeführt, die ein akkreditiertes Trinkwasserlabor in den Jahren 1997–2019 untersucht hatte.

**Ergebnisse:**

Es konnten 16.060 Proben analysiert werden. 75,36 % dieser Proben waren unterhalb der quantitativen Bestimmungsgrenze. Der Median der quantifizierbaren Proben lag in jedem betrachteten Jahr oberhalb des zukünftigen Grenzwertes für Blei im Trinkwasser. Es konnte kein Effekt der letzten Übergangsfrist von 10 Jahren (2003–2013) festgestellt werden.

**Diskussion:**

Auch wenn keine systematischen Untersuchungen zur Bleibelastung in deutschen Kommunen vorliegen, ist die Thematik von hoher Aktualität. Bleiexposition durch Trinkwasserinstallationen ist ein vollständig vermeidbares Gesundheitsrisiko, dass jedoch nur bei konsequenter Durchsetzung der geltenden Regeln durch die Gesundheitsbehörden reguliert werden kann. Diese sind hierzu personell, materiell und finanziell ausreichend auszustatten.

## Abstract (en)

**Background:**

Lead pipes have been used in drinking water installations for a long time. Only since 1973 have their use in new buildings been strongly discouraged; nevertheless, they are still found in old buildings. Furthermore, lead-containing alloys are still used today in components such as fittings. This results in an avoidable pollution of drinking water. The health significance of this pollution is acknowledged by a reduction of the currently valid limit value from 10 µg/l to 5 µg/l by the new EU drinking water directive passed in 2020. This directive provides for a transition period of 15 years.

**Objectives:**

The relevance of a stricter limit value for lead in drinking water will be evaluated based by the results of routine analyses, and the necessary public health measures to prevent exposure of vulnerable groups will be formulated.

**Material and methods:**

A retrospective analysis of routine samples from the city of Bonn, which an accredited drinking water laboratory had examined in the years 1997–2019, was performed.

**Results:**

Of the 16,060 samples analyzed, 75.36% were below the quantitative limit of quantification. The median of the quantifiable samples was above the future limit for lead in drinking water in each year considered. No effect of the last transition period of 10 years (2003–2013) could be detected.

**Discussion:**

Even though no systematic studies on lead pollution in German municipalities are available, the subject is still highly topical. Lead exposure from drinking water installations is a completely avoidable health risk, but it can only be regulated by consistent enforcement of the applicable rules by the health authorities. The health authorities must be adequately equipped in terms of personnel, material, and financial resources.

## Einleitung

Blei (lateinisch Plumbum, englisch „lead“, Symbol *Pb*, Ordnungszahl 82) ist ein giftiges Schwermetall, welches in der Erdkruste mit einem Gehalt von ca. 0,0018 % meist in Form von Erzen (als Sulfide, Carbonate u. a., selten auch in reiner Form) vorkommt. Die stabilen Bleiisotope ^206^Pb, ^207^Pb und ^208^Pb stellen die jeweiligen Endnuklide der 3 radioaktiven Zerfallsreihen dar.

Bleiverbindungen werden durch eine Vielzahl natürlicher, aber vor allem anthropogener Prozesse in die natürliche Umwelt freigesetzt und verteilt. Blei hat toxische Effekte auf Mikroorganismen, Pflanzen, Tiere und Menschen und kann bioakkumulieren [1, 2].

Anthropogene Quellen für Blei waren und sind der Abbau, das Verhütten, Raffinieren und Recyceln, aber aktuell auch der vielfältige Einsatz in verschiedensten industriellen und gewerblichen sowie anderen zivilen und militärischen Bereichen (Bleiakkumulatoren, Strahlenabschirmung, Apparatebau, Legierungsbestandteil, Bleifarben, Lötlot, Geschosse, Schrot u. v. a. m.). Über den bis 1973 erlaubten Einsatz reiner Bleirohre oder auch von bleilässigen verzinkten Eisenrohren im Bereich der Trinkwasserinstallation von Gebäuden kam und kommt es immer noch zu nennenswerten Belastungen des Trinkwassers [3].

Aufgrund seiner chemisch-physikalischen Eigenschaften wie guter Verformbarkeit, niedrigen Schmelzpunkts und hoher Korrosionsbeständigkeit [1, 4] ist Blei bereits seit dem Altertum ein häufig verwendeter Werkstoff für Wasserleitungen, u. a. wurde es für innerstädtische römische Wasserverteilungssysteme verwendet, aber auch für Überlandleitungen, wie z. B. bei Lyon [5]. Der römische Architekt und Autor Vitruvius riet in seinem Werk *De architectura libri decem* (ca. 30 v. Chr.) bereits von der Nutzung von Bleirohren aus gesundheitlichen Gründen und wegen des schlechten Geschmacks ab [6], was darauf schließen lässt, dass Rohre dieses Materials zu jener Zeit in größerem Umfang eingesetzt wurden. Die gesundheitsschädliche Bedeutung von Bleirohren im Trinkwassersystem gilt nach heutigem toxikologischen Kenntnisstand als erwiesen.

Blei gilt nach internationaler Einschätzung als „major public health concern“ und ist damit von großer Bedeutung für die öffentliche Gesundheit: Im Jahre 2017 wurden weltweit ca. 1.060.000 Todesfälle und 24.400.000 DALYs (*Disability Adjusted Life Years* = durch Behinderung beeinträchtigte Lebensdauer) durch Bleiexposition verursacht [3].

Für die Allgemeinbevölkerung steht die Aufnahme von Blei über Lebensmittel und Trinkwasser im Vordergrund [2]. Diese wird für Deutschland mit etwa 0,5–30 Mikrogramm pro Kilogramm (µg/kg) Körpergewicht und Tag angegeben [7]. Lebensmittel, die mehr als 80 % zur täglichen Bleiaufnahmemenge beitragen können, variieren in ihrem Bleigehalt: Tierische Lebensmittel, vor allem Innereien, können bis zu 1000 µg/kg enthalten, abhängig von dem Bleigehalt des Futters. Pflanzliche Lebensmittel können bis zu 600 µg/kg enthalten [7].

Die Bleiaufnahme über den Pfad Trinkwasser ist von verschiedenen Faktoren abhängig: Länge der Trinkwasserinstallation, Stagnationszeit des Wassers in den Leitungen, pH-Wert, Wasserhärte und anderen Wassereigenschaften.

Die Resorptionsrate von oral aufgenommenem Blei wurde lange auf etwa 8–10 % bei Erwachsenen und bis zu 50 % bei Kindern geschätzt [2, 7]. In der neueren Literatur wird für Erwachsene sogar eine Resorptionsrate von 20–70 % angegeben; für Kinder kann sie altersabhängig noch höher als 70 % liegen (Säuglinge, Ungeborene; [1]).

Im Blut wird resorbiertes Blei überwiegend an Hämoglobin gebunden [10] und schnell im Körper verteilt [2]. Zielorgane sind im toxikologischen Sinne das Gehirn, die Leber und die Nieren. In Knochen und Zähnen ersetzt nicht ausgeschiedenes Blei das vorhandene Calcium und akkumuliert dort mit Halbwertszeiten von mehreren Jahren [2, 3, 8].

Obwohl akute Intoxikationen ab einer Blutkonzentration von 1 µg/l (Referenzwert Erwachsene < 250 µg/l) vorkommen [9], stehen bei der trinkwasserbedingten Bleiexposition die chronischen Effekte im Vordergrund.

Zu diesen zählen hämatologische Effekte (Anämie, Ikterus), neurologische Effekte (Kopfschmerzen, Depression, Krampfanfälle, Muskelschwäche), gastrointestinale Effekte (Magenschmerzen und Koliken) sowie Nierenschädigungen [3]. Ob Blei auch negative immunologische Effekte hat, ist gegenwärtig unklar [10]. Anorganisches Blei wird von der Internationalen Agentur für Krebsforschung (IARC) der Weltgesundheitsorganisation (WHO) als „wahrscheinlich krebserregend“ (Gruppe 2A) eingestuft [11].

Vor allem für Ungeborene, Säuglinge und Kleinkinder ist Blei von besonderer toxikologischer Bedeutung, da Blei die Plazentaschranke überwinden und zu Schädigungen des blutbildenden Systems sowie des Nervensystems führen kann. Darüber hinaus kann es zur Minderung der Intelligenzentwicklung kommen. Bei schwangeren Frauen kann Blei aus den Knochen z. B. aus früheren Intoxikationen ins Blut remobilisiert werden und so das Ungeborene schädigen.

Zur Therapie von akuten und chronischen Bleivergiftungen eignen sich Chelatbildner [12]. Da die Therapie der chronischen Bleivergiftung langwierig ist und auch Residuen vorkommen [1], gilt die Expositionsprophylaxe (als Primärprävention) schließlich als geeignete und vordringliche Maßnahme. Für den Trinkwasserbereich bedeutet das ein konsequentes Substituieren von bleihaltigen Trinkwasserinstallationen (Bleirohren, bleihaltigen verzinkten Eisenleitungen) sowie von bleilässigen Bauteilen wie Fittings, Rohrverbindern, Armaturen, Wasserzählern oder Probennahmeventilen [13, 14].

Bleirohre wurden bereits im 19. Jahrhundert im Süden des Deutschen Reichs aus präventivgesundheitlichen Gründen verboten, während man im Norden die Nutzung aufgrund der technischen Vorteile weiter gestattete [13]. Auch spielte die – zum Teil bis heute erhaltene – Vorstellung einer angeblich bleiundurchlässigen „Kalkschutzschicht“ eine Rolle, welche sich leider als unrichtig erwies. Der endgültige Verzicht auf Bleirohre wurde allerdings erst in der DIN 2000 (Deutsche Industrie-Norm) von 1973 formuliert: „Die Verwendung von Bleirohren ist gesundheitlich bedenklich, da sich Blei lösen und im Trinkwasser anreichern kann. Für neue Trinkwasserleitungen sollen daher Bleirohre nicht mehr verwendet werden“ [15].

**Figure 1:**
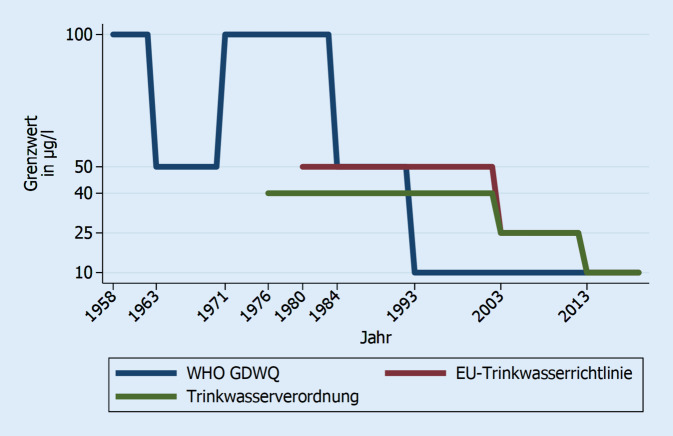

Für Bestandsanlagen bis 1973 wurden parallel die internationalen und nationalen Anforderungen bezüglich der Leit- bzw. Parameterwerte für Blei im Trinkwasser zunehmend verschärft (Abb. 1).

Die letzte Überarbeitung der Leitlinien für Trinkwasserqualität der WHO (*Guidelines for Drinking-Water Qualitiy, GDWQ*) fand im Jahre 2017 statt. Der seit 1993 bestehende Leitwert für Blei von 10 µg/l wurde beibehalten, jedoch nun als „provisorischer Leitwert“ bezeichnet, der sich aus der technischen und analytischen Machbarkeit ergibt, und explizit nicht aus Gründen des Gesundheitsschutzes [16]. Hintergrund dieser Änderung in der Argumentation ist die Tatsache, dass der vorläufig tolerierbare wöchentliche Aufnahmewert PTWI (*Provisional Tolerable Weekly Intake*) im Jahr 2011 durch den Gemeinsamen Sachverständigenausschuss für Lebensmittelzusatzstoffe (JECFA) der WHO und der Ernährungs- und Landwirtschaftsorganisation der Vereinten Nationen (FAO) revidiert wurde, da ein PTWI von 25 µg/kg Körpergewicht pro Woche im Mittel mit einer Beeinträchtigung des Intelligenzquotienten von 3 Punkten und einem systolischen Blutdruckanstieg von 3 mm Hg einherging [17]. Da aus toxikologischer Sicht weder ein Schwellenwert noch ein PTWI-Wert festgelegt werden kann [13, 18], müssen alle vernünftigerweise ergreifbaren Maßnahmen zur Senkung der wöchentlichen Bleiaufnahme, besonders bei Risikogruppen wie Kindern und Schwangeren, im Sinne des sogenannten ALARA-Prinzips (*As Low As Reasonably Achievable* *=* so niedrig wie vernünftigerweise erreichbar) umgesetzt werden.

Die novellierte Trinkwasserrichtlinie der Europäischen Union (EU), die über eine Neufassung der Trinkwasserverordnung in Deutschland rechtlich bindend wird, wurde am 23.10.2020 vom Rat der Europäischen Union beschlossen und wurde am 15.12.2020 vom Europäischen Parlament verabschiedet [19]. Bezugnehmend auf das ALARA-Prinzip wurde eine weitere Senkung des Bleiparameterwertes auf 5 µg/l festgelegt mit einer Übergangsfrist von 15 Jahren nach Inkrafttreten der Richtlinie [19].

Zur adäquaten und EU-einheitlichen Erfassung von Trinkwasserbelastungen wurde das Verfahren „Zufallsstichprobe (Z-Probe)“ festgelegt [20]. Diese ermöglicht lediglich eine grobe Übersicht, ob in einem Versorgungsgebiet Auffälligkeiten hinsichtlich einer erhöhten Nachweisfrequenz von Blei vorliegen, jedoch keine Aussage, ob eine Grenzwertüberschreitung an einer einzelnen Entnahmestelle vorliegt [14, 20], weshalb hierfür die „gestaffelte Stagnationsbeprobung (S-Probe)“ angewendet werden soll.

In dieser Arbeit soll anhand einer retrospektiven Auswertung (1997–2019) von Routineproben eines akkreditierten Trinkwasserlabors untersucht werden, welche Bleikonzentrationen in den letzten 20 Jahren in Trinkwasserproben der Stadt Bonn auftraten. Es soll aufgezeigt werden, wie sich eine weitere Übergangsfrist von mehreren Jahren bei Verschärfung des Grenzwertes für Blei im Trinkwasser auswirken kann. Ferner soll diskutiert werden, welche Maßnahmen aus Public-Health-Perspektive notwendig und hinreichend erscheinen, um eine Gesundheitsgefährdung abzuwenden.

## Methoden

Zur Beurteilung der Relevanz von Blei im Trinkwasser wurden die Daten des akkreditierten Trinkwasserlabors des Institutes für Hygiene und Public Health des Universitätsklinikums Bonn herangezogen. Hierzu erfolgte im Februar 2020 eine Datenbankabfrage für den Zeitraum 1997–2019. In die Betrachtung wurden alle Untersuchungen eingeschlossen, die den Parameter „Bleikonzentration“ im Medium „Trinkwasser“ enthielten.

Die Probenanalyse erfolgte bis März 2010 mit Graphitrohr-Atomabsorptions-Spektrometrie, danach mit Massenspektrometrie mit induktiv gekoppeltem Plasma (ICP-MS, Aginelt Techniologies, 7700 Series).

Die Auswertung der Rohdaten erfolgte qualitativ sowie quantitativ mittels Stata 15.1 IC für Windows. Die gemessenen Bleikonzentrationen wurden mit dem jeweils geltenden Grenzwert verglichen und qualitativ mittels Balkendiagrammen nach Jahren grafisch dargestellt, wobei 4 Klassen gebildet wurden:

- kleiner als die quantitative Bestimmungsgrenze,
- kleiner als der geplante Grenzwert von 5 µg/l,
- größer als der geplante Grenzwert von 5 µg/l, aber kleiner als der jeweils geltende Grenzwert,
- größer als der jeweils geltende Grenzwert.

Die Darstellung erfolgte mittels absoluter Zahlen sowie in Relation bezogen auf die Gesamtprobenzahl pro Jahr.

Quantifizierbare Proben, also Werte oberhalb der quantitativen Bestimmungsgrenzen, wurden im Anschluss als Boxplots nach Jahren aufgetragen.

## Ergebnisse

**Figure 2:**
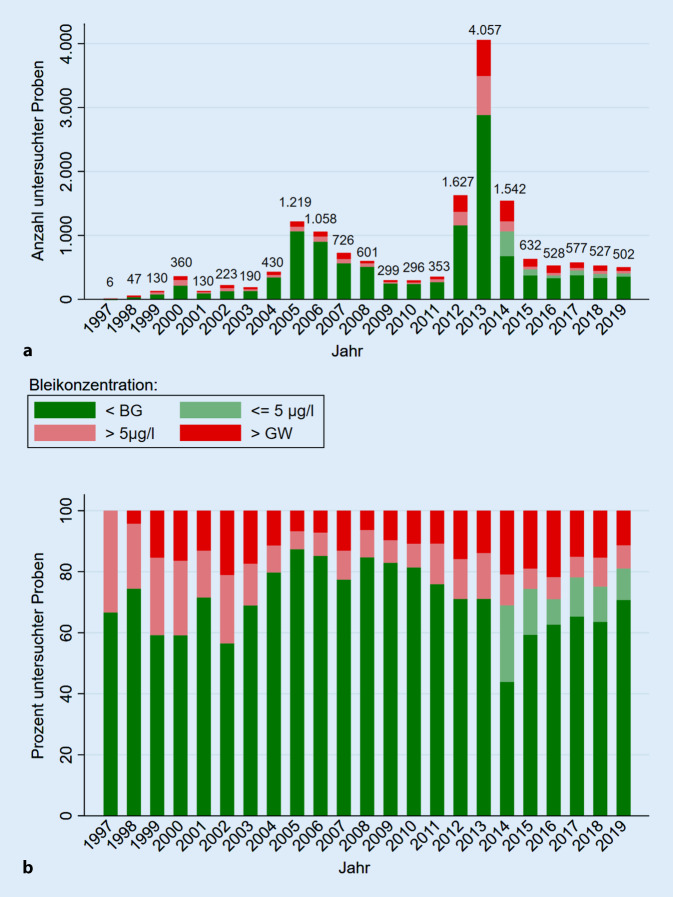

Es wurden insgesamt 16.060 Trinkwasserproben identifiziert, bei denen ein auswertbarer Bleibefund vorlag. Diese verteilen sich über die Jahre 1997 bis 2019, wie in Abb. 2 dargestellt. Es zeigen sich Spitzen bei der Probenanzahl in den Jahren 2000, 2004/2005/2006 sowie 2012/2013/2014. Seit 2015 liegt die Anzahl untersuchter Proben relativ stabil bei etwa 550 Proben pro Jahr. In Tab. 1 ist die Verteilung der Proben nach Probennehmer und Probenart dargestellt.

**Table 1:** 

	Probenanzahl	Prozentualer Anteil (%)
*Probenahme*		
Einsender selbst	4035	25,12
Akkreditiert durch Labor	12.025	74,88
*Art der Probenahme*		
Z‑Probe	10.245	63,79
S0-Probe	427	2,66
S1-Probe	2143	13,34
S2-Probe	2159	13,44
Sonstige/Unbekannt	1086	6,76

*Z‑Probe* Zufallsstichprobe, *S‑Proben* gestaffelte Stagnationsbeprobung (*S0* Nullprobe nach Herstellung einer Temperaturkonstanz, *S1* erster Liter nach Stagnation, *S2* zweiter Liter nach Stagnation)

Unter Berücksichtigung der jeweils geltenden quantitativen Bestimmungsgrenzen und Grenzwerte ergibt sich eine Verteilung der Analyseergebnisse, wie in Abb. 2 dargestellt.

Die Bestimmungsgrenze lag bis 2013 bei 5 µg/l. Der Anteil der Analyseergebnisse unterhalb der Bestimmungsgrenze lag in den Jahren von 1997–2013 bei 75,36 %, der Anteil beanstandungswürdiger Proben (>als der jeweils geltende Grenzwert) lag bei 12,24 %.

Ab 2014 sank die Bestimmungsgrenze auf 2 µg/l, sodass seitdem eine weitere Unterscheidung der quantifizierbaren Probenergebnisse unterhalb des geplanten Grenzwertes möglich ist. Der Anteil der Analyseergebnisse unterhalb dieser neuen Bestimmungsgrenze lag in den Jahren von 2014 bis 2019 bei 56,87 %, der Anteil beanstandungswürdiger Proben lag bei 18,15 %. Weitere 8,43 % aller untersuchten Proben wären bei Zugrundlegen des geplanten Grenzwertes (EU-Trinkwasserrichtlinie 2020 [19]) von 5 µg/l zu beanstanden gewesen. Die höchste gemessene Bleikonzentration im beobachteten Zeitraum betrug 27.000 µg/l im Jahr 2013.

**Figure 3:**
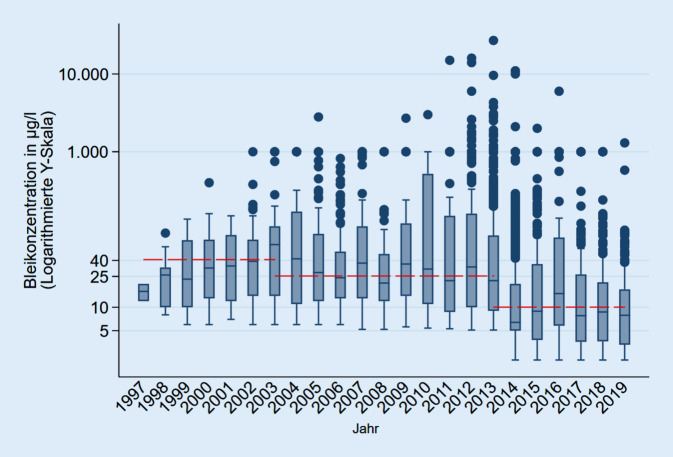

Betrachtet man ausschließlich die quantifizierbaren Probenergebnisse oberhalb der Bestimmungsgrenzen, ergibt sich ein Bild, wie in Abb. 3 dargestellt. Es zeigen sich mediane Bleikonzentrationen um den jeweils geltenden Grenzwert mit Ausreißern, die den Grenzwert um ein Vielfaches überschreiten. Die medianen Werte liegen seit 2014 durchgehend über dem geplanten Grenzwert von 5 µg/l.

In der Detailbetrachtung der Jahre 2003 bis 2013 zeigt sich eine gleichbleibende Verteilung von Probenergebnissen in Bezug auf den geltenden Grenzwert, sodass ein Effekt der Übergangsfrist an den vorliegenden Daten nicht erkennbar ist. Im Gegenteil zeigt der deutliche Anstieg der Untersuchungszahlen im Jahre 2013, dass viele Untersuchungen erst wenige Monate vor Ende der Übergangsfrist angefordert wurden.

## Diskussion

Anhand der ausgewerteten Bonner Routineproben der Jahre 1997–2019 lässt sich zeigen, das Blei im Trinkwasser auch heute noch ein relevantes Problem darstellt. Durchschnittlich 18,15 % der untersuchten Proben seit 2014 wiesen Bleikonzentrationen auf, die (zum Teil sehr weit) über dem jeweils geltenden Grenzwert lagen. Legt man den geplanten neuen Grenzwert zugrunde, erhöht sich dieser Anteil noch einmal, sodass dann über ein Viertel aller untersuchten Proben nicht den Anforderungen an Trinkwasser entsprächen.

### Anforderungen an Trinkwasser und Trinkwasserversorgungsanlagen

Trinkwasser kann als wichtigstes Lebensmittel nicht substituiert werden [21]. Aus diesem Grunde werden besondere Anforderungen an die Trinkwasserbeschaffenheit gestellt. Trinkwasser muss „so beschaffen sein, dass durch seinen Genuss oder Gebrauch eine Schädigung der menschlichen Gesundheit insbesondere durch Krankheitserreger nicht zu besorgen ist. Es muss rein und genusstauglich sein“ (§ 4 Abs. 1 TrinkwV [22]).

Um diesem Anspruch gerecht zu werden, müssen einerseits a) bei der Wassergewinnung, der Wasseraufbereitung und der Wasserverteilung die allgemein anerkannten Regeln der Technik eingehalten werden, andererseits muss b) Trinkwasser den chemischen, mikrobiologischen und radiologischen Anforderungen der TrinkwV entsprechen:

- Obwohl seit 1973 keine Bleirohre mehr in Trinkwasserinstallationen verbaut werden sollen [15] und auch die aktuelle Positivliste metallener Werkstoffe im Kontakt mit Trinkwasser Blei als Werkstoff ausschließt, sind bleihaltige Werkstoffe für Armaturen, Rohrverbinder, Apparate und Pumpen und darin enthaltene Bauteile weiterhin zugelassen [23]. Eine Kontamination von Stagnationswasser mit Blei ist beim Vorhandensein von bleihaltigen Trinkwasserleitungen bzw. der Verwendung entsprechender Armaturen usw. abhängig von Konstruktion und Alter der Trinkwasserinstallation, chemischer und physikalischer Beschaffenheit des Trinkwassers und Betriebsbedingungen (vor allem der Stagnationszeit) nicht auszuschließen [14]. Daher gilt bereits bei einer Betriebsunterbrechung von über 4 h, dass das Wasser vor Gebrauch bis zur Temperaturkonstanz ablaufen soll [24].
- Wie eingangs dargestellt, ergeben sich der gegenwärtige sowie der zukünftige Grenzwert von 10 µg/l bzw. 5 µg/l allein aus Überlegungen zur technischen Machbarkeit [16]. Aus toxikologischer Sicht müsste der Grenzwert wegen des Nichtvorhandenseins eines unbedenklichen Schwellenwerts noch niedriger sein [13, 18, 22, 25].

Unsere quantifizierbaren analysierten Proben wiesen teils so hohe Konzentrationen auf (Abb. 3), dass eine reine Kontamination durch Armaturen allein unwahrscheinlich ist. Die massive Bleibelastung von Trinkwasser mit Konzentrationen bis zu 27.000 µg/l spricht eher für das Vorhandensein von Bleirohren oder massive Korrosion bleihaltiger Anlagenteile in einigen Bereichen des Einzugsgebietes. Doch auch schon bleihaltige Armaturen können dazu führen, dass der aktuelle bzw. künftige Grenzwert nicht mehr gehalten werden kann. Damit entsprechen die betroffenen Trinkwasseranlagen nicht den allgemein anerkannten Regeln der Technik und dürften so nicht weiter betrieben werden. Eine Gesundheitsgefährdung durch den Konsum dieses Wassers ist gegeben.

### Trinkwasser für besonders vulnerable Gruppen

Die in der TrinkwV festgelegten Grenzwerte gelten implizit für den „gesunden Verbraucher“ [26] als Durchschnitt der Bevölkerung, können aber spezifische Anforderungen an die Wasserqualität für besonders vulnerable Personen nicht vollständig regulativ abdecken. Für den Bereich mikrobiologischer Kontamination mit bspw. Legionellen, Pseudomonaden u. a. wasserübertragenen Erregern ist diese Situation insbesondere in Krankenhäusern relevant und wird dort durch strengere Anforderungen und Maßnahmen [27–29] kontrolliert.

Im Falle von Blei befinden sich erhöht vulnerable Personengruppen wie Ungeborene, Säuglinge und Kleinkinder jedoch auch im häuslichen Umfeld. Entsprechende Verhaltensempfehlungen existieren zwar [30], Substitution und technische Maßnahmen zur Verminderung der Konzentrationen toxischer Stoffe sind jedoch vorzuziehen.

### Aktualität der Gefahr

Anhand unserer ausgewerteten Daten lässt sich zeigen, dass die Relevanz von Grenzwertüberschreitungen für Blei im Trinkwasser nicht an Aktualität verloren hat. Systematische Studien zur Bleibelastung innerhalb der einzelnen Kommunen liegen nicht vor, daher kann die generelle Relevanz des Themas in Deutschland nicht valide abgeschätzt werden. Allerdings liegt eine orientierende nichtrepräsentative Untersuchung der Stiftung Warentest aus dem Jahr 2004 vor, die neben Bonn und Frankfurt am Main vor allem die neuen und die nördlichen Bundesländer als mögliche Hotspots darstellt [31]. Für die Stadt Bonn zeigen die hiesigen Daten für den Zeitraum 1997–2019, dass die Bleibelastung im Trinkwasser nach wie vor ein aktuelles Problem darstellt. Es ist, mit Ausnahme von Frankfurt am Main und den süddeutschen Bundesländern, davon auszugehen, dass auch andere Kommunen Deutschlands mit erhöhten Bleiwerten in Trinkwasser konfrontiert sein könnten.

### Bleisanierung am Beispiel der Stadt Frankfurt am Main

Durch das Stadtgesundheitsamt Frankfurt wurde 1997 das „Frankfurter Bleiprojekt“ initiiert, um die ab 2003 bzw. 2013 geltenden Grenzwerte für Blei im Trinkwasser einhalten zu können [32]. Die Umsetzung erfolgte rechtzeitig vor Inkrafttreten des Grenzwerts 2003. Anhand unserer Daten lässt sich zeigen, dass die 10-jährige Übergangsfrist bis 2013 in anderen Gebieten Deutschlands, hier in der Stadt Bonn, nicht helfen konnte, das Problem als relevant zu erachten und zu lösen. Aus den Erfahrungen des Frankfurter Projektes hinsichtlich Kosten, Zeit- und Personalaufwands sowie der Rechtsunsicherheit aufgrund der unklaren europäischen Regelung lassen sich folgende Herausforderungen ableiten:

- es besteht ein Handlungszwang,
- es besteht faktisch ein Sanierungszwang,
- es besteht ein Zeitproblem,
- es besteht ein Problem der reproduzierbaren Probenahme.

Während der letzte Punkt mit der Beschreibung der Z‑Probe sowie vor allem der gestaffelten Probenahme [14, 20] suffizient gelöst ist, haben die übrigen Punkte heute unverändert Bestand und werden mit Inkrafttreten der neuen EU-Trinkwasserrichtlinie in ihrer Relevanz weiter zunehmen. Eine Selbstlösung des Problems nur allein durch Festlegung einer Übergangsfrist scheint in Anbetracht der Auswertung der letzten Grenzwertverschärfung für Bonn sehr unwahrscheinlich, wenn das Problem nicht aktiv angegangen wird.

### Forderungen für die Zukunft

Aus Sicht der Autoren sind spätestens für die Umsetzung der neuen EU-Trinkwasserrichtlinie, besser aber ab sofort, folgende Forderungen zu stellen und durch die jeweiligen Adressaten umzusetzen:

1. Bundeseinheitliches Verbot von bleihaltigen Materialien innerhalb von Trinkwasserinstallationen sowie klare Formulierung der entsprechenden rechtlichen Möglichkeiten zur bundeseinheitlichen Durchsetzung dieses Verbotes. Zuständig sind die jeweiligen Gesundheitsämter. Hierzu sind klare Positionierungen des Umweltbundesamtes sowie des Bundesamtes für Risikobewertung erforderlich.
2. Übernahme der Verantwortung zur Durchsetzung des Bleiverbotes durch die Gesundheitsämter. Eine Verantwortungsdelegation auf organisatorische Maßnahmen (dauerhaftes Spülen) oder rechtliche Maßnahmen durch Mieter (Klage gegen Vermieter) ist ethisch fragwürdig und verbietet sich.
3. Ausreichend frühe und ausreichend umfangreiche Kommunikation durch die Behörden gegenüber den Vermietern und Mietern. Die positiven Erfahrungen aus Frankfurt zeigen, dass nur mit einer konsequenten Um- und Durchsetzung der notwendigen Maßnahmen vom ersten Tag der Übergangsfrist an ein erfolgreiches Risikomanagement inklusive einer Sanierungskontrolle betrieben werden kann.

Die Hauptlast bei der Erfüllung dieser Forderungen tragen die Gesundheitsämter; daher ist dringlich zu fordern, dass diese entsprechend ihrer wichtigen Funktion zur Aufrechterhaltung der öffentlichen Gesundheit personell, materiell und finanziell besser ausgestattet werden. Insofern ist der Pakt für den Öffentlichen Gesundheitsdienst, auf den sich die Bundeskanzlerin und die Regierungschefinnen und Regierungschefs der Länder im September 2020 geeinigt haben, ein wichtiger und notwendiger Schritt [33].
